# Reduction of unnecessary antibiotic days in a level IV neonatal intensive care unit

**DOI:** 10.1017/ash.2022.33

**Published:** 2022-03-28

**Authors:** Dipen P. Vyas, Vilmaris Quinones-Cardona, Margaret A. Gilfillan, Megan E. Young, Kimberly A. Pough, Alison J. Carey

**Affiliations:** 1 Pediatrics, St. Christopher’s Hospital for Children, Drexel University College of Medicine, Philadelphia, Pennsylvania; 2 Department of Pharmacy, St. Christopher’s Hospital for Children, Philadelphia, Pennsylvania; 3 Microbiology and Immunology, Drexel University College of Medicine, Philadelphia, Pennsylvania

## Abstract

**Objective:**

Antibiotics are widely prescribed in the neonatal intensive care unit (NICU) and duration of prescription is varied. We sought to decrease unnecessary antibiotic days for the most common indications in our outborn level IV NICU by 20% within 1 year.

**Design and interventions:**

A retrospective chart review was completed to determine the most common indications and treatment duration for antibiotic therapy in our 39-bed level IV NICU. A multidisciplinary team was convened to develop an antibiotic stewardship quality improvement initiative with new consensus guidelines for antibiotic duration for these common indications. To optimize compliance, prospective audit was completed to ensure antibiotic stop dates were utilized and provider justification for treatment duration was documented. Multiple rounds of educational sessions were conducted with neonatology providers.

**Results:**

In total, 262 patients were prescribed antibiotics (139 in baseline period and 123 after the intervention). The percentage of unnecessary antibiotic days (UAD) was defined as days beyond the consensus guidelines. As a balancing measure, reinitiation of antibiotics within 2 weeks was tracked. After sequential interventions, the percentage of UAD decreased from 42% to 12%, which exceeded our goal of a 20% decrease. Compliance with antibiotic stop dates increased from 32% to 76%, and no antibiotics were reinitiated within 2 weeks.

**Conclusions:**

A multidisciplinary antibiotic stewardship team coupled with a consensus for antibiotic therapy duration, prescriber justification of antibiotic necessity and use of antibiotic stop dates can effectively reduce unnecessary antibiotic exposure in the NICU.

Antibiotics are the most commonly prescribed medication in the neonatal intensive care unit (NICU).^1,2^ Although neonatal sepsis is associated with both considerable mortality and morbidity,^3^ early antibiotic exposure is also associated with adverse outcomes in preterm neonates including chronic lung disease, late-onset sepsis, necrotizing enterocolitis, periventricular leukomalacia, severe retinopathy of prematurity, and poor long-term neurodevelopmental outcomes.^4–7^ Unnecessary antibiotic use in the NICU can lead to separation of the neonate from the parent due to NICU admission, delay in breast feeding, increased healthcare costs,^8^ antibiotic resistance,^9^ and invasive candidiasis.^10^ Finally, early antibiotic exposure is also linked to increased incidence of atopy, allergic disorders, asthma, and childhood weight gain.^11–13^

Critically ill neonates are frequently initiated on antibiotics because of their high risk of sepsis. However, literature is limited regarding effective antibiotic stewardship strategies to reduce unnecessary antibiotic days for the most medically complex, critically ill neonate in the level IV NICU. In review of our local practices, we noted a lack of uniform duration of antibiotic therapy, inconsistent diagnostic criteria for infections, and fear of treatment failure which contributed to unnecessary antibiotic days. A multidisciplinary NICU antibiotic stewardship team was developed to address these inconsistencies and to increase awareness of the importance of antibiotic stewardship in the neonatal population. We sought to decrease unnecessary antibiotic days (UAD) for the most common antibiotic indications in our NICU by 20% within 1 year.

## Methods

### Setting

This quality improvement project was initiated at St. Christopher’s Hospital for Children’s NICU, a 39-bed level IV regional perinatal center in Philadelphia, Pennsylvania. Approximately 250 neonates are admitted to our NICU annually, and all patients are outborn and transferred from regional NICUs. Admitted infants frequently require advanced surgical and pediatric subspecialty services.

### Baseline period and identification of primary drivers for unnecessary antibiotic prescription

**Fig. 1.**
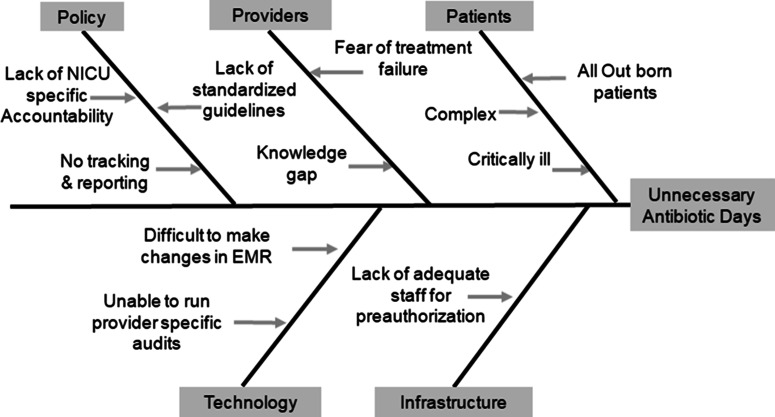
Contributing factors for unnecessary antibiotic days. A fishbone diagram was created to outline the most important reasons for unnecessary antibiotic days.

**Fig. 2.**
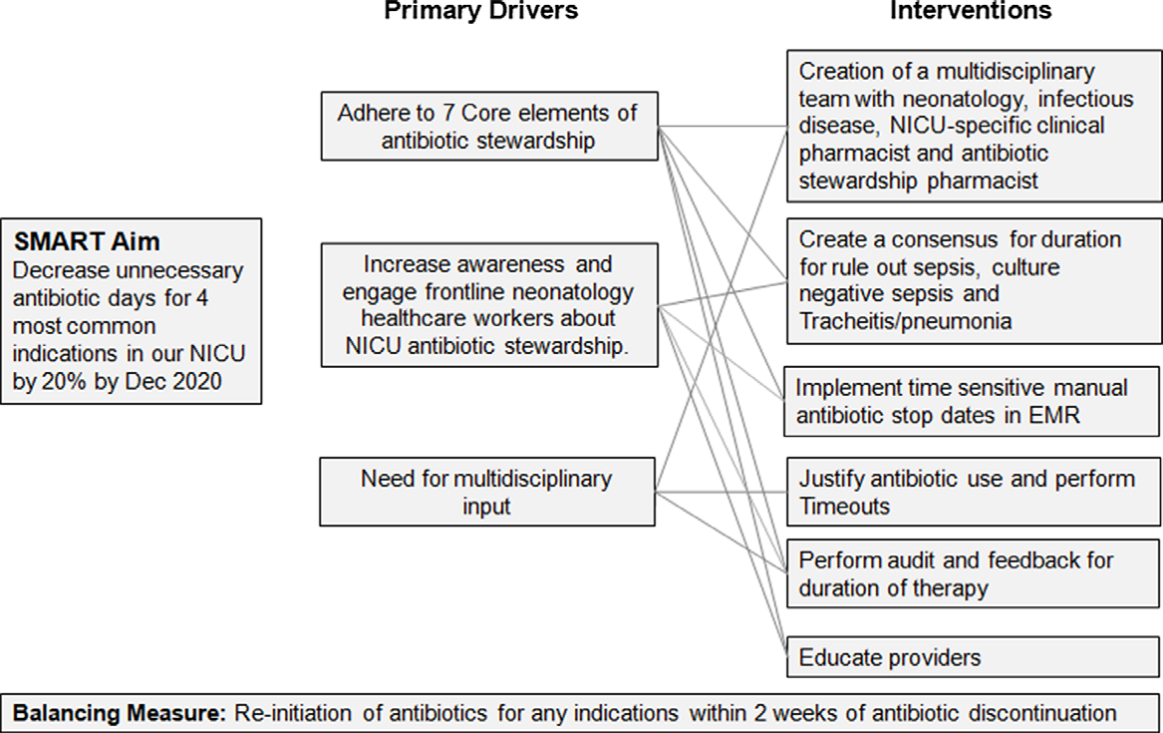
Primary drivers for unnecessary antibiotic days. A diagram was created of the primary drivers which led to unnecessary antibiotic days. Based on these drivers, appropriate interventions were devised to improve antibiotic usage.

Baseline data from January 1, 2019, to December 31, 2019, were collected retrospectively. Through analysis of retrospective data and focus group discussions with providers, the primary reasons for UADs were identified and a fishbone diagram was created (Fig. 1). The most common reason for prolonged antibiotic therapy was fear of treatment failure and complexity of the patient population, such as those on extracorporeal membrane oxygenation or therapeutic hypothermia. In addition, despite having a hospital-wide antibiotic stewardship team, with pharmacy and infection prevention expertise, there was no coordinated effort focused on neonatal antibiotic stewardship. Therefore, a NICU-specific multidisciplinary antimicrobial stewardship team was needed. A team of a neonatal fellow, neonatal nurse practitioner, neonatology attending, infectious disease attending, NICU-specific clinical pharmacist and an antimicrobial stewardship pharmacist was formed and convened in December 2019 to review the baseline data and develop targeted interventions. The following primary drivers were identified to achieve a reduction in UADs: (1) to ensure adherence to the 7 core elements of Center for Disease Control and Prevention (CDC) hospital antibiotic stewardship program (ie, hospital leadership commitment, accountability, pharmacy expertise, action, tracking, reporting, and education)^14^; (2) to increase awareness and engage frontline neonatology healthcare workers about NICU antibiotic stewardship; and (3) to address the need for multidisciplinary input (Fig. 2).

### Interventions

**Fig. 3.**
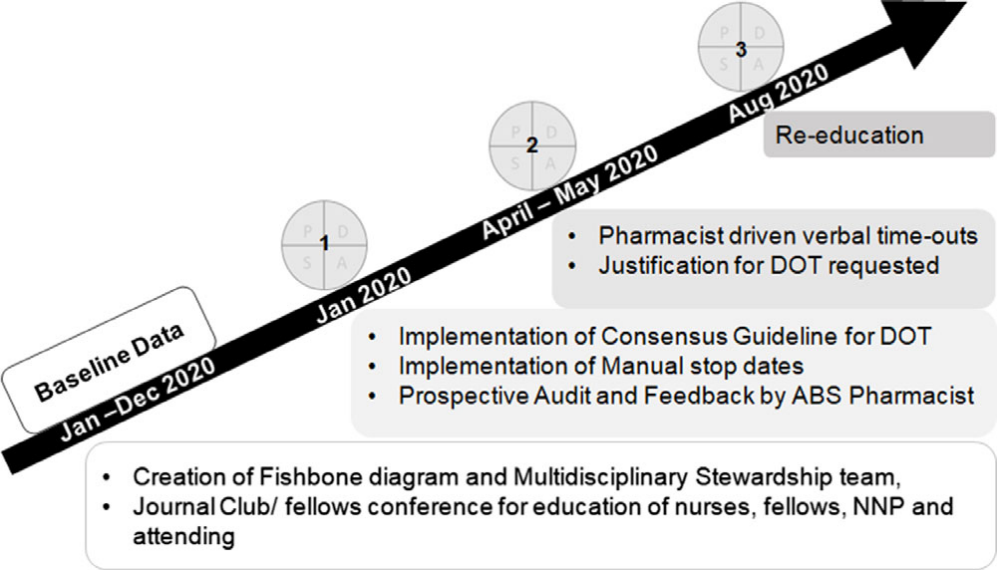
Timeline for plan–do–study–act (PDSA) cycles and interventions. There was a baseline period to collect data on antibiotic days and then a series of 3 PDSA cycles, with the outlined interventions.

Over the course of the study period, 5 specific interventions to address these drivers were implemented to achieve the primary aim. We used the Institute for Healthcare Improvement’s Model for Improvement for this initiative and plan–do–study–act (PDSA) cycles to assess our progress (Fig. 3).^15^ Discussions were inconsistent regarding justification for the initiation (indication), continuation, and duration of antibiotic use during rounds, with tremendous inter-prescriber variability. To increase awareness and engage NICU providers about antibiotic stewardship, a consensus guideline for therapy duration was developed, manual antibiotic stop dates were implemented, justification of antibiotic use was requested, and a series of educational sessions were conducted. In our level IV unit, a NICU-specific clinical pharmacist is present on rounds 5 days a week who encouraged justification and verbal timeouts during rounds. In addition, the antibiotic stewardship pharmacist provided prospective audit and feedback.

### Consensus guideline for duration of therapy

In the retrospective data review, the most common indications for antibiotic prescription were (1) rule out sepsis, (2) culture-negative sepsis, and (3) respiratory tract infection (tracheitis and pneumonia). In addition, we noted wide variation in antibiotic duration for each indication between providers. During the baseline period, treatment to rule out sepsis had a duration of 2–3 days, treatment for culture-negative sepsis had a duration of 5–14 days, treatment for tracheitis had a duration of 5–7 days, and treatment for pneumonia had a duration of 7–10 days, based on provider preference. Thus, based on this wide variation, interventions focused on the development of consensus guidelines for these (ie, most common) indications of antibiotic use. Available evidence was reviewed to develop appropriate therapy duration for these indications, and a consensus was determined by the division and presented during an educational conference on January 14, 2020, (Table 1). Based on this consensus, we adopted standardized duration of therapy of 48 hours to rule out sepsis, 5 days for culture-negative sepsis and tracheitis, and 7 days for uncomplicated pneumonia.

**Table 1.** Timeline and Topics Discussed During the Educational Sessions

Date	Topic
11/26/2019	Education Conference reviewing “Reducing unnecessary antibiotic use in the neonatal intensive care unit (SCOUT): a prospective interrupted time-series study”^16^
1/14/2020	Research conference—Discussion of new consensus guidelines for antibiotic duration: Suspected sepsis/rule out sepsis: 48 hours Culture negative sepsis: 5 days Tracheitis: 5 days Uncomplicated pneumonia: 7 days
10/6/2020	Journal club: Shortening the antibiotic course for the treatment of neonatal coagulase-negative staphylococcal sepsis—Fine with 3 days?^17^
1/5/2021	External lecturer—Antibiotic stewardship in the NICU
1/26/2021	Evidenced based medicine—Culture-negative sepsis

### Manual antibiotic stop dates

Our retrospective analysis revealed that antibiotics were unnecessarily prolonged because there was a delay in manual discontinuation of antibiotics. Automatic duration of therapy or a hard stop on specific antibiotics were considered but could not be implemented because the electronic health record (EHR) system did not have this feature. Therefore, to avoid delay in antibiotic discontinuation, antibiotic orders were required to have a manually entered stop date (Supplementary Fig. 1). This intervention began on January 1, 2020.

### Justification of antibiotic use and verbal time outs

Providers were encouraged to have discussions during daily rounds about indications and duration for continued antibiotic use. Documentation of this discussion in the daily progress note was requested. If there was a decision to prescribe a patient an antibiotic therapy course longer than consensus guidelines, justification was required in the daily progress note. Starting April 1, 2020 (PDSA 2), the clinical pharmacist performed a verbal time-out on rounds 24–48 hours after antibiotic initiation for every patient prescribed antibiotics. Time-outs included defining the indication for antibiotic use, appropriate duration of therapy and potential narrowing of antibiotic therapy. This process was performed daily until all questions were documented in the progress note.

### Education of the providers

Several educational sessions were held to discuss consensus guidelines, to reinforce the importance of antibiotic stop dates, and to request documentation of justification of antibiotics for all the neonatology fellows, neonatology attending physicians, neonatal nurse practitioners, and nurses. Fellow educational conferences were used as opportunities for education of the neonatal fellows and attending physicians (Table 1). In addition, small group discussions were performed with neonatal fellows and rotating residents in the NICU at the start of their rotation to update them on the interventions. Antibiotic choice was not a focus of this initiative.

### Prospective audit and feedback

Antibiotic stewardship, pharmacist-driven, prospective audit and feedback began on January 14, 2020. The antibiotic stewardship pharmacist was responsible to perform a daily screen of antibiotic duration and stop dates in the EHR for any patients on antibiotics >48 hours. Antibiotic duration was considered prolonged if it was longer than the consensus guidelines. If there was prolonged antibiotic duration or was no stop date, the stewardship pharmacist called the neonatology fellow, pediatric resident physician, or advanced practitioner in the NICU to provide real-time feedback. Ultimately, the final decision for the antibiotic duration was at the primary team’s discretion.

### Outcomes

The primary outcome was percentage of patients with UADs per 2-week interval. UADs were defined as therapy duration beyond the consensus guidelines. All antibiotic orders were reviewed for compliance with inclusion of a stop date. Total number of stop dates ordered was tracked as a percentage of total number of patients initiated on antibiotics each month. For percentage of justification of antibiotic use, progress notes of only those patients who had UADs and 5 other randomly selected progress notes were reviewed retrospectively every month for documentation of why antibiotics were initiated, indication, duration of therapy, and reason therapy was longer than the consensus guidelines if there were UADs.

The antibiotic stewardship pharmacist performed prospective audit and feedback for prolonged therapy duration, and the total number of feedback sessions was tracked as a percentage of total number of patients initiated on antibiotics each month. The most frequent reason that the focus group (the neonatal attending, fellows, and nurse practitioners) cited to not decrease duration of antibiotics was failure of therapy. Therefore, we tracked reinitiation of antibiotics for any indication within 2 weeks of onset of antibiotic prescription as a balancing measure.

### Statistical analysis

Descriptive analysis was conducted for gestational age, birth weight, any antibiotic exposure and antibiotic use for each indication. A descriptive analysis and Pearson χ^2^ test were performed with statistical package for social sciences using SPSS version 24 software (IBM, Armonk NY). The outcome, process and balancing measures were displayed using statistical process control (SPC) P and X bar and S charts. QI-Macros 2020 software was used to analyze and generate SPC charts. Centerlines and 3-Δ (delta) control limits were applied when at least 8 consecutive points were above or below the center line, when 1 or more data points fell beyond the control limits, or when 6 consecutive points comprised a trend in either direction.^18,19^ Centerlines were adjusted based on detection of special cause signal.^20^ Ethical aspects of the interventions and studying of interventions was reviewed by local institutional review board and was exempt from consent by the Internal Review Board of Drexel University College of Medicine (IRB 2012008285, February 11, 2021).

## Results

### Antibiotic prescription in the NICU

During the study period, 514 neonates were admitted to the NICU. Of these, 250 were admitted during the baseline period (January 1, 2019, to December 31, 2019) and 264 were admitted during the postintervention period (January 1, 2020, to February 28, 2021). Charts were reviewed for exposure to at least 1 antibiotic prior to discharge, noted as antibiotic exposures. In total, 139 (55%) of 250 neonates were exposed to at least 1 antibiotic in the baseline period compared to 123 (46%) of 264 in the postintervention period. Of these antibiotic-exposed neonates, 90 (65%) of 139 in the baseline period and 86 (70%) of 123 in the postintervention period were treated with antibiotics for the most common indications (ie, rule out sepsis, culture negative sepsis, and respiratory tract infection) (Table 2). Birth weight and gestational age were comparable between the 2 periods. We observed significant variability of the age of the neonate at the time antibiotics were initiated, based on the diagnosis.

**Table 2.** Patient Demographics

		Baseline (1/1/19–12/31/19)	After the Intervention (1/1/20-2/28/21)	*P* Value
Variable		No. (%)	No. (%)	
Total patients		250	264	…
Antibiotic exposure^a^		139 (55)	123 (46)	…
Antibiotic exposure for targeted indications^b^		90 (65)	86 (70)	…
Birth weight	<750 g	11 (12)	8 (9)	0.38
	751–1,500 g	12 (13)	12 (13)	
	1,500–2,500 g	25 (28)	18 (19)	
	>2,500 g	42 (47)	54 (59)	
Gestational age	<28 weeks	16 (18)	18 (19)	0.47
	28–34 weeks	21 (23)	15 (16)	
	>34 weeks	53 (59)	60 (64)	
				Age at Initiation of Antibiotics Median Days
Diagnoses No. (%)^c^	Rule-out sepsis	84 (39)	89 (35)	1 (early onset) 37 (late onset)
	Culture-negative sepsis	27 (12)	27 (11)	1 (early onset) 52 (late onset)
	Tracheitis	23 (11)	20 (8)	159
	Pneumonia	9 (4)	12 (5)	81
	Necrotizing enterocolitis	11 (5)	26 (10)	20
	Culture positive sepsis	5 (2)	11 (4)	44
	Urinary tract infection	12 (6)	6 (2)	61
	Perioperative prophylaxis	17 (8)	38 (15)	…
	Other diagnoses^d^	29 (13)	28 (11)	…
	Total	217 (100)	257 (100)	…

a Antibiotic exposure: total number of patients exposed to at least 1 antibiotic divided by total patients admitted during the period.

b Antibiotic exposure for most common indications: total number of patients exposed to antibiotics for most common indications divided by total antibiotic exposure

c Total instances of antibiotic initiation for specific indication divided by total number of instances of antibiotic initiation.

d Other diagnoses included prophylaxis for medical indication like asplenia, renal, etc, congenital syphilis, skin abscess, neurosurgical hardware infection, mediastinitis, *Clostridium difficile*, gastrointestinal abscess.

### Reduction of unnecessary antibiotic days after implementation of consensus guidelines, antibiotic stop dates and improved justification

**Fig. 4.**
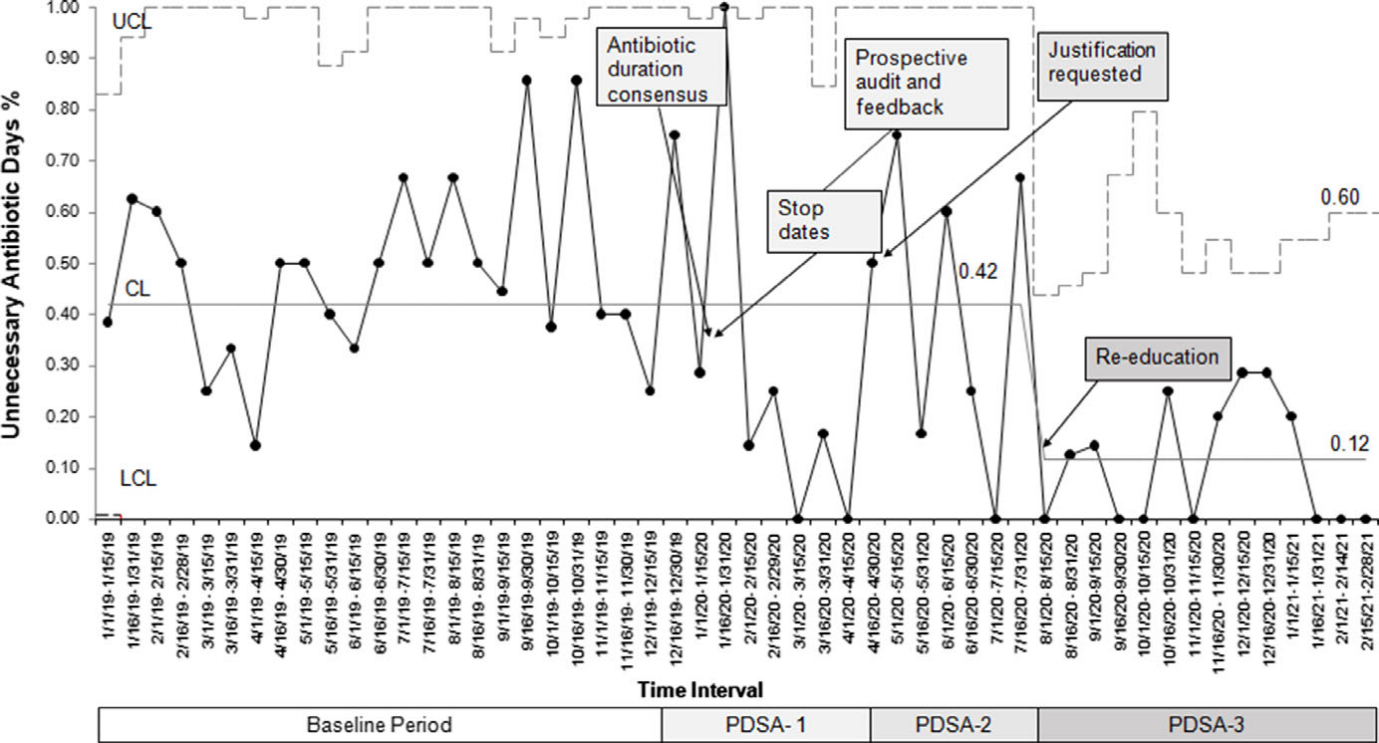
Improvement in the percentage of patients exposed to unnecessary antibiotic days. To track the effectiveness of the outlined interventions, a p-chart was created of the biweekly percentage of patients with unnecessary antibiotic days. Note. UCL, upper control limit; CL, center line; LCL, lower control limit.

After 3 PDSA cycles (Fig. 3), we noted a special cause variation, defined by 8 consecutive points below the centerline, with a decrease in the average percentage of patients with UAD from 42% to 12% (Fig. 4). Special cause variation was also noted with 1 data point above the upper control limit during the 2-week interval of January 16, 2020–January 31, 2020. During this time, only 3 patients were on antibiotics, which was fewer than during other intervals. Also, 2 of these patients had a complicated clinical course with worsening acute on chronic pulmonary hypertension, and the third patient was on extracorporeal membrane oxygenation, which prompted the primary team to treat longer based on clinical presentation. This data point was included in the center-line calculation to avoid a false reduction in the percentage of patients with UAD.

**Fig. 5.**
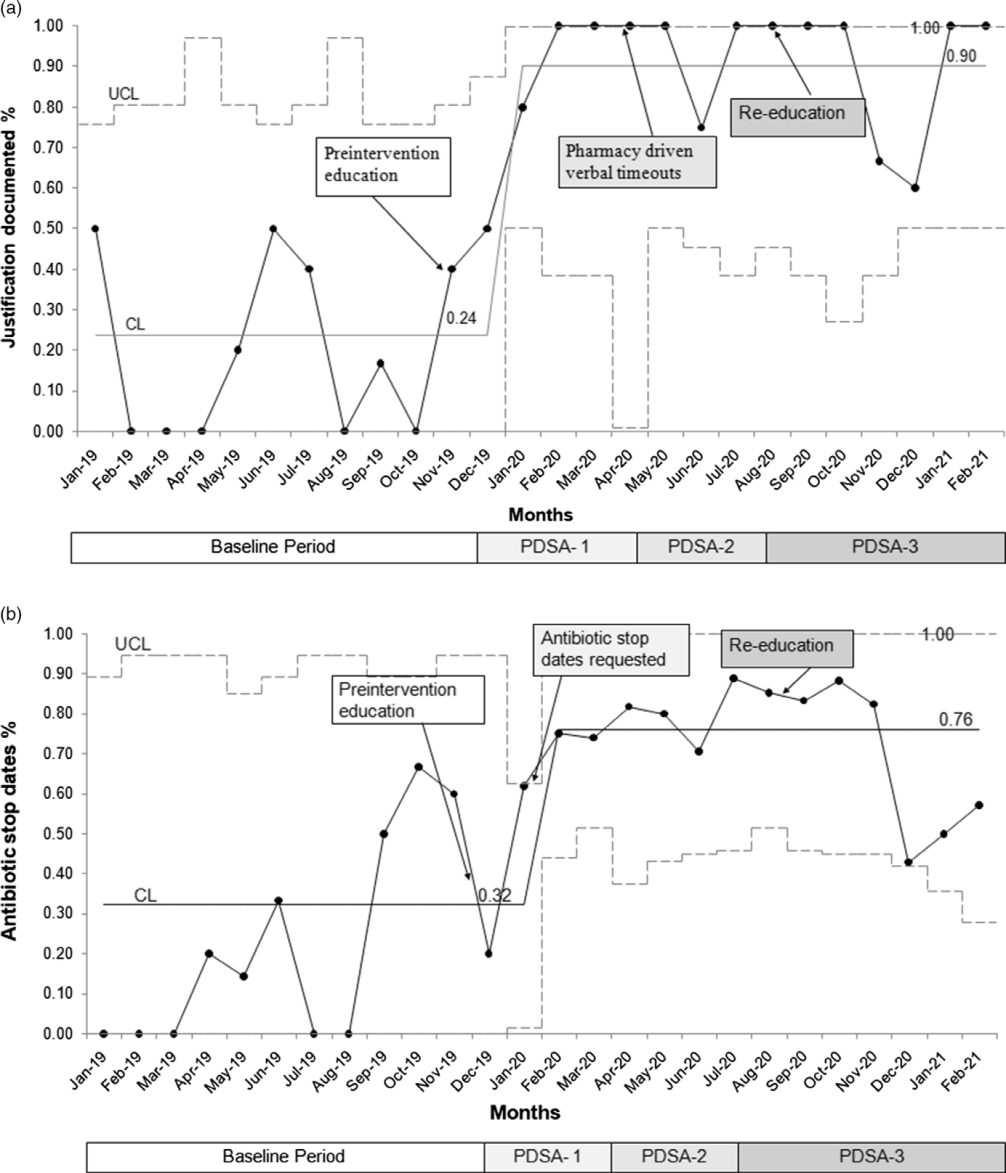
Process measures. The p-chart for (A) monthly documentation justifying antibiotic use in the daily progress notes and (B) percentage of antibiotic stop dates used per month.

We observed special cause variation in the percentage of antibiotic stop dates used, which increased from 32% to 76% (Fig. 5A). Documentation of justification of antibiotic use improved, with an increase from 24% to 90% and special cause variation (Fig. 5B). Prospective audit and antibiotic stewardship feedback was performed in 20% of total patients on antibiotics by antibiotic stewardship pharmacist during the postintervention period. Importantly, no change occurred in the average percentage of reinitiation of antibiotics per month.

## Discussion

In a level IV NICU with a medically complex outborn population, there can be considerable variability in antibiotic prescription duration. Therefore, standardized guidelines for antibiotic duration must be developed for appropriate antimicrobial stewardship in this unique population. Here, a comprehensive approach for antibiotic stewardship to safely reduce UAD at a level IV NICU was achieved through the creation of a NICU-specific multidisciplinary antibiotic stewardship team, standardized duration of therapy for the most common antibiotic indications, antibiotic stop dates and prospective audit and feedback.

Although antibiotic stewardship strategies have been described in the neonatal population, literature is limited regarding stewardship initiatives specifically in NICUs that care for the most critically ill neonates. Antibiotic stewardship interventions have focused on shortening the duration of antibiotic therapy,^16,21–23^ reduction of initiation of antibiotic therapy,^21,24,25^ and infrastructural antibiotic stewardship program implementation.^21,24,26,27^ Here, we employed 2 of these key components: reduction of the duration of antibiotic therapy through consensus guidelines and the creation of a NICU-specific antibiotic stewardship program. Reduction of initiation of antibiotic therapy, such as the use of an early-onset, sepsis-risk calculator, can reduce antibiotic exposure in infants >35 weeks gestational age in the delivery hospital setting.^28,29^ However, this model is less practical in our medically complex term or preterm infants, who frequently have been initiated on antibiotics by the referring institution.

Identification of strategies that could be both safely and effectively applied to a level IV NICU population was of particular importance in this antibiotic stewardship initiative. Several strategies have been applied to reduce the duration of antibiotics in high-acuity inborn NICUs, including antibiotic hard stops,^22,23^ standardized duration of therapy for common indications,^16^ and prospective audit and feedback.^14^ Here, we applied these interventions in an outborn level IV NICU with some unit-specific modifications. We encountered infrastructure difficulties in making changes in the EHR to implement automatic antibiotic stop dates. Therefore, provider education to manually enter stop dates at the time of antibiotic prescription was completed. Although manual entry of stop dates was more labor intensive, this approach was effective in reducing the percentage of patients with UAD in our NICU, as demonstrated by others,^16^ and this strategy could be utilized by other centers with similar EHR restrictions.

The development of a consensus guideline for duration of therapy targeted at the indications mostly commonly used in the NICU was an important component of effectively reducing UAD. Developing evidence-based guidelines with infectious disease input was key to engage the neonatologists and create consensus. Given the clinical severity and complexity of our patients, our interventions centered on a team-based approach to provider education with real-time discussions and feedback, while affording neonatologists the autonomy to initiate and select antibiotics of their choice. Ultimately, this autonomy improved acceptance of the consensus guidelines and drove culture change.

Similar to central-line–associated bloodstream infection reduction initiatives,^30^ a successful component of our initiative was requesting daily documentation to justify antibiotic prescription. The clinical pharmacist conducted a verbal time-out during daily rounds if antibiotics remained necessary and discussed opportunities to narrow therapy and planned duration. This particular intervention is consistent with the tenet of accountability in the CDC Core Elements of Hospital Antibiotic Stewardship Programs.^14^ A recent multicenter national quality improvement project of the Vermont Oxford Network reduced antibiotic use by 34% with improved adherence to several of the CDC Core Elements.^31^ Similarly, we were successful in reducing the percentage of patients with UADs by 70% with our multipronged approach.

This study had several limitations. The initiative was conducted at a single-center NICU. Therefore, generalizability may be limited to level IV NICUs with a similar patient population and a full complement of resources, such as a dedicated clinical pharmacist present during daily rounds. Furthermore, the scope of this initiative was limited to the most common indications for antibiotic use. Culture-proven infection of blood and urinary tract were rare during this epoch. Our consensus guidelines focused on treatment for either culture-negative sepsis or positive respiratory cultures, which frequently reflect colonization. Further work should explore treatment duration for culture-proven infections in the most vulnerable patients with immature immune systems, especially in the first weeks after birth.

A multidisciplinary antibiotic stewardship team coupled with focused interventions including the use of standardized duration of therapy, antibiotic justification, stop dates, and prospective audit and feedback can effectively and safely reduce UAD in an outborn level IV NICU. Future PDSA cycles will expand the breadth of this initiative to other antibiotic indications and will facilitate strategies that focus on reducing antibiotic initiation to further decrease UAD in our NICU.

## Supplementary material

For supplementary material accompanying this paper visit https://doi.org/10.1017/ash.2022.33. click here to view supplementary material
