# Supplementary figures and images for: Reduction of unnecessary antibiotic days in a level IV neonatal intensive care unit
Source: Antimicrob Steward Healthc Epidemiol. 2022 Mar 28;2(1):e50. doi: 10.1017/ash.2022.33 (PMC9726496; doi:10.1017/ash.2022.33)

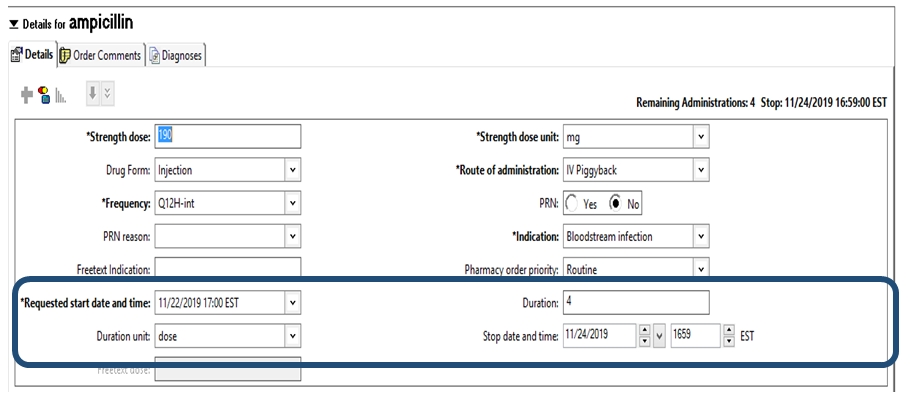

Supplement: Supplementary file 1 [file S2732494X2200033Xsup001.tiff]
